# Histidine-based copper tetrapeptides as enantioselective catalysts for aldol reactions†

**DOI:** 10.1039/c8ra06814c

**Published:** 2018-10-02

**Authors:** Begum Sharifa Zaithun, AbdulMalek Emilia, Tahir Mohamed Ibrahim Mohamed, Crouse Karen Anne, Abdul Rahman Mohd Basyaruddin

**Affiliations:** Integrated Chemical BioPhysics Research, Faculty of Science, Universiti Putra Malaysia 43400 UPM Serdang Selangor Malaysia; Department of Chemistry, Faculty of Science, Universiti Putra Malaysia 43400 UPM Serdang Selangor Malaysia basya@upm.edu.my

## Abstract

Copper(ii)-peptides are widely used as industrial catalysts such as in the aerobic oxidation of organic molecules, formation of new C–H bonds and in the azide–alkyne cycloaddition reaction. The length of peptides and the effect of adding copper metal into peptides were questioned in their field of applications. Five novel histidine-based tetrapeptides with the sequences HAAD (P1), HAFD (P2), HAVD (P3), AGHD (P4) and PGHD (P5) were synthesized using the solid phase peptide scheme and analysed with high performance liquid chromatography (HPLC) and liquid chromatography-mass spectrometry (LC-MS) with percentage purities as high as 99.5%. All the peptides were positively charged (+1) and the molecular weight calculated from *m*/*z* values of MS results coincided with the theoretical molecular weight of the peptides. Copper(ii)-peptides derived from these peptides and copper(ii) acetate monohydrate (CuP1–CuP5) in a 1 : 2 ratio was synthesised, purified and characterised by ultraviolet-visible spectroscopy (UV-Vis), ultraviolet-fluorescence spectroscopy (fluorescence) and fourier transform infrared spectroscopy (FTIR), circular dichroism spectroscopy (CD) and optical rotation polarimetry. It provided the necessary information on the secondary structure and the successful binding of copper(ii) to the specific amino acids, hence leading to the putative geometry of copper(ii)-peptides and the difference in the chirality of amino acids, peptides and copper(ii)-peptides. The catalytic activities of the synthesised complexes were evaluated. CuP1 & CuP3 catalysed both the asymmetric aldol reactions with high enantioselectivity of *p*-nitrobenzaldehyde with cyclohexanone (% ee = 87.3 & 80.3, respectively) and of *p*-anisaldehyde with cyclohexanone (% ee = 95.5 & 90.9, respectively).

## Abstract 2

P5 with the sequence H_2_N-PGHD-CONH.

## Graphical abstract

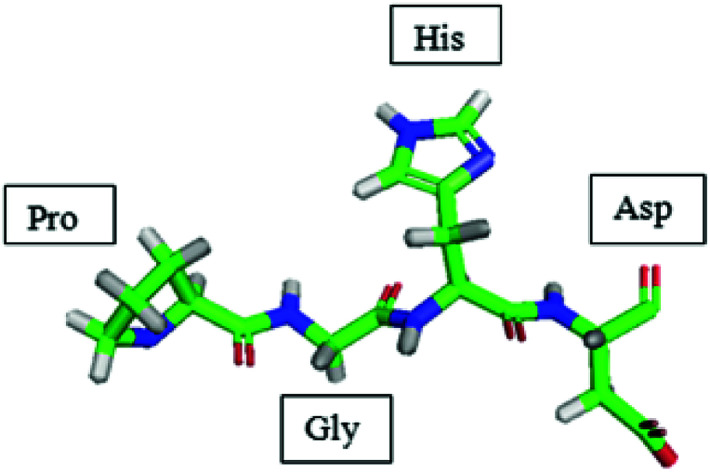

## 1. Introduction

The aldol and aldol condensation reactions are versatile organic reactions in the construction of the C–C bond.^1^ In these reactions, two carbonyl compounds are utilized, one electrophile and one nucleophile. Aldol reactions have the ability to construct larger molecules from smaller ones, some by cyclization effects, with the control of stereochemistry in the form of enantiomers and diastereomers of aldol adducts.^2^ For the past few decades, enzymes have been popular for use as practical catalysts in organic synthesis. For example, aldo keto reductase and Type I aldolases have been used as catalysts for aldol reactions. However, their low activity in the form of percentage yields and enantiomeric excess encouraged researchers to design and modify small molecules based on the enzyme's active site.^1^

Natural and synthetic peptides have been used not only in the medicinal field to combat diseases but also in the industrial field especially for the purpose of organic synthesis. Tetrapeptides are preferred over longer chain peptides (greater than 8 amino acids) for the synthesis of copper(ii)-peptides in terms of cost effectiveness and to combat chemical and thermal stability issues. Copper(ii)-peptides are synthesized as a tool for industrial application to replace longer chain peptides.^3^ It is advantageous in terms of cost and stability to incorporate metals into short chain peptide due to the extra ionic and covalent bonds as a replacement of hydrogen bonds in long chain peptides that have a definite secondary structure.^4^

Metallo di- and tri-peptides have also been reported to be unfavourable as they were unstable and had no definite geometry.^4^ To form a stable copper(ii)-peptide, the metal should bind to more than one-metal binding amino acid, like histidine, aspartic acid, glutamic acid, cysteine and methionine. The latter two amino acids are not preferred for biological applications as the sulphur in these amino acids makes them more toxic with their side products (H_2_S is produced instead of H_2_O).^5^ The number and location of the carboxylate groups from the side chains of aspartate and glutamate amino acids is important in the binding of metal as it contributes to the negative charge of the copper(ii)-peptide making it an ideal platform for metal binding.^6^ Preferred binding sites of metal to side chains of amino acids are the first and third amino acid of peptides.^7^

The versatility of histidine coordination favours transition metal bindings and many researches were conducted to characterize the binding interactions of metal to histidine.^8,9^ Cun *et al.* reported that zinc(ii) and iron(ii) had a higher preference to bind with histidine in the presence of acidic amino acids such as aspartic or glutamic acid in the sequence.^9^ The carboxylate acid side chain group together with histidine's imidazole rings forms the facial triad motif with the transition metal that facilitates the catalytic activity of a reaction.^10^

List and co-workers (2000) investigated the catalytic effects of asymmetric aldol reaction between acetone and *p*-nitrobenzaldehyde using various different primary amino acids as catalysts. Single amino acid, histidine, tyrosine, phenylalanine and valine catalyzed the above said aldol reaction to produce <10% of the aldol adduct.^11^ Meanwhile, in 2011, Huang and his research team used dipeptides with valine and proline as one of the amino acids in their sequence for direct asymmetric aldol reaction. This resulted in high percent enantioselectivity with low yield of aldol adducts.^12^ Similar results were observed when tripeptides were used as catalysts for the same direct asymmetric aldol reaction. The adduct yield was observed to be <30% while the amount of enantiomeric excess was >80%.^13^

Herein, tetrapeptides and copper(ii)-derived tetrapeptides were synthesized, purified and characterized by ultraviolet-visible, ultraviolet-fluorescence, Fourier transform infrared spectroscopies and optical rotation studies by polarimetry to prove the presence of copper(ii) in the tetrapeptide. They were then tested for their catalytic activities in direct asymmetric aldol reactions between *p*-nitrobenzaldehyde/*p*-anisaldehyde and cyclohexanone.

## 2. Experimental section

### 2.1 Materials

All the chemicals and solvents used in these experiments were of analytical reagent grade and further purification of these solvents were not needed. The amino acids and HCTU were purchased from GL Biochem (Shanghai) Ltd. The organic solvents were purchased from J. T. Baker, Fisher Scientific and Merck.

### 2.2 Physicochemical characterization

Melting point of peptides and copper(ii)-peptides were measured using digital melting point apparatus. Plastic plates of thin layer chromatography (TLC) were pre-coated with silica gel 60 *F*_254_ (0.2 mm thickness), Merck brand (fluorescent). High performance liquid chromatography-ultraviolet detector (HPLC-UV) analysis were carried out using Waters HPLC (Binary HPLC pump 1525 and UV-Waters 2489) employing gradient system (deionized water with 0.1% trifluoroacetic acid (TFA) and acetonitrile with 0.1% TFA) with 0.5 mL min^−1^ flowing through reversed phase C_18_ column XBridge (4.6 × 250 mm). Liquid chromatography-mass spectroscopy (LC-MS) analysis were conducted using Agilent 1290 Infinity LC system coupled to Agilent 6520 accurate-mass Q-TOF mass spectrometer with dual ESI source with the same gradient system and flow rate as HPLC. The column used was Agilent Zorbax 300SB-C18 Narrow-Bore RR (2.1 × 100 mm × 3.5 μm). Peptides and their copper complexes were analysed using Perkin Elmer spectrum 100 with wavenumbers ranging from 4500–450 cm^−1^. Secondary structure and optical rotation of peptides and Cu(ii)-peptides in phosphate buffer (KH_2_PO_4_) were studied using the JASCO P1050 spectrometer and JASCO P-2000 polarimeter, respectively.

### 2.3 Synthesis of peptides

Five tetrapeptides (P1: HAAD, P2: HAFD, P3: HAVD, P4: AGHD and P5: PGHD) were designed using Lomets^14^ and PyMol software.^15^ They were synthesized following the solid phase peptide synthesis scheme (SPPS) employing the rink amide resin protected by 9-fluoromethylcarbonyl (Fmoc).^17^ The general synthesis of tetrapeptide; P1 is shown in ESI Scheme 1.† The remaining peptides with different amino acids were synthesized following the same scheme (ESI Scheme 1†).

### 2.4 Synthesis of copper(ii)-tetrapeptides

Copper(ii) acetate monohydrate; Cu(CH_3_COO)_2_·H_2_O solution (1.6 mmol, 5 mL) was added dropwise into the pre-prepared peptide solution (0.8 mmol, 5 mL) with constant stirring at 40 °C (optimized condition). The resulting solution was stirred until a precipitate formed for an approximate of 2 hours. Color changes of the resulting solution were also observed.^16^ The pH of the solution was maintained at pH = 6 throughout the binding process. The precipitate was vacuum-filtered and washed with cold deionized water and dried with acetone. The product was purified through a crystallization process and the melting point was measured. The copper(ii)-peptides were characterized as mentioned in Section 2.2. The copper-peptides were labeled following the peptides sequence: CuP1: Cu–P1–Cu, CuP2: Cu–P2–Cu, CuP3: Cu–P3–Cu, CuP4: Cu–P4–Cu and CuP5: Cu–P5–Cu.

### 2.5 Catalysts in aldol reactions

**Scheme 1:**
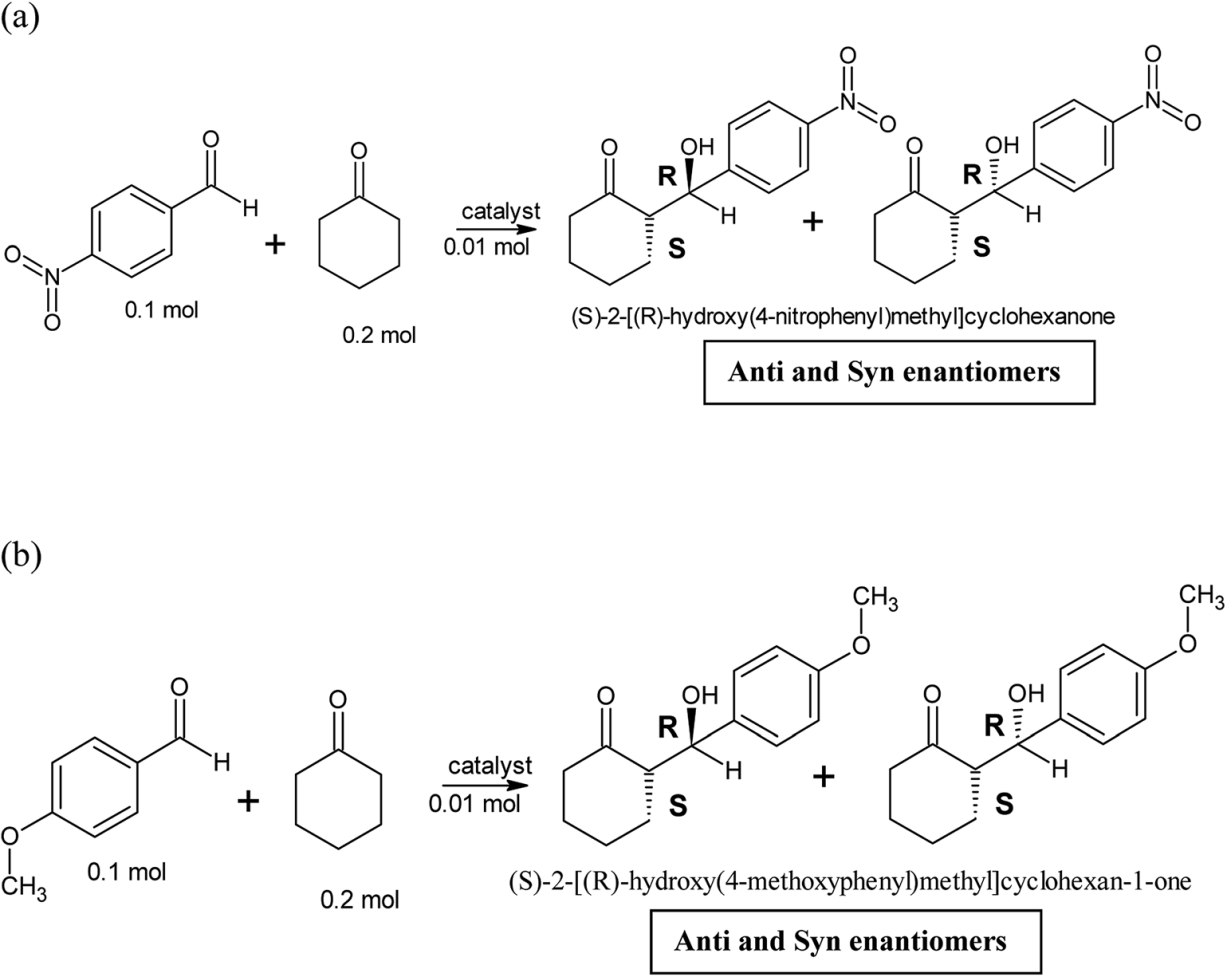
(a) Aldol reaction between *p*-nitrobenzaldehyde (electron withdrawing group) and cyclohexanone (reaction 1). (b) Aldol reaction between *p*-anisaldehyde (electron donating group) and cyclohexanone (reaction 2).

Two different aldol reactions were carried out using three different substrates, *p*-nitrobenzaldehyde and cyclohexanone and *p*-anisaldehyde and cyclohexanone as shown in Scheme 1. The peptides and copper(ii)-peptides (catalyst, 0.1 mol, 10 mol%) were dissolved in a mixture of isopropanol and water (1 : 1 v/v ratio). NMP (*N*-methyl-2-pyrrolidone), a base, was added to the catalyst to neutralize it to pH = 7. Cyclohexanone (2 mol) was added to the mixture and left to stir for 20 minutes. The appropriate aldehyde (1 mol) was weighed and added to the cyclohexanone containing catalyst and left to stir at room temperature for 48 hours.^1^ TLC was recorded every 24 hours. This reaction was carried out without any catalyst (negative control) and employing l-proline (positive control).

## 3. Results and discussion

### 3.1 Design of tetrapeptides

**Fig. 1:**
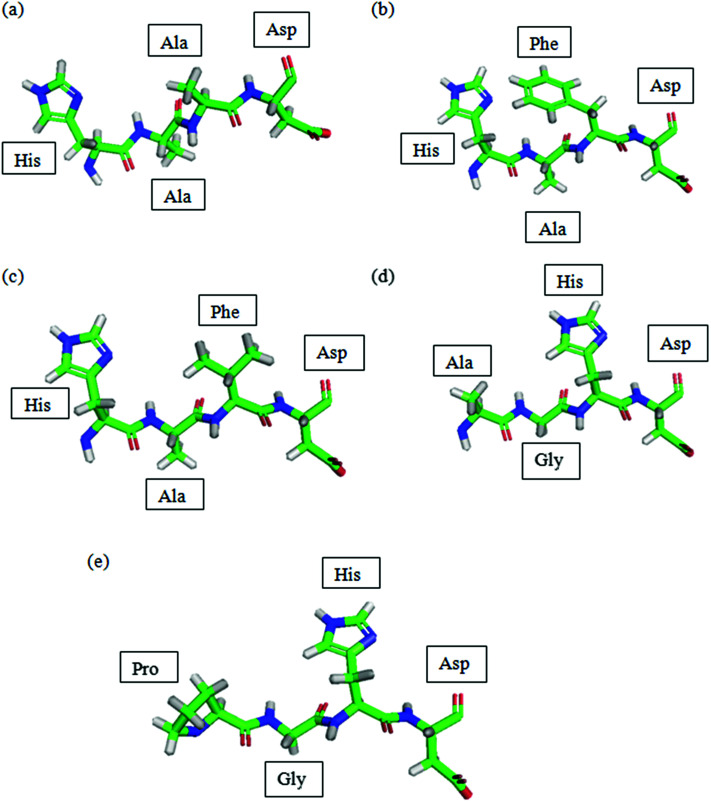
PDB of peptides predicted by Lomets and viewed using PyMol. (a) P1; (b) P2; (c) P3; (d) P4; (e) P5. Colour code denoted by: green: carbon; blue: nitrogen; red: oxygen; grey: hydrogen.

The peptides designed were viewed using PyMol as shown in Fig. 1.

### 3.2 HPLC & LCMS analysis of tetrapeptides

The crude peptides were analyzed using HPLC to obtain the percent purity of peptides using the C_18_ reverse phase column employing the optimised gradient system. Several gradient systems were tested for each peptide and the best results were utilized. TFA was added to the mobile phase as the ion pairing reagents as the silica packing in the column could cause strong interactions with peptide, hence the flow of the peptide through the column would be made difficult if it adhered to the column. TFA in the mobile phase pairs with the peptide and elutes out.^18^ The purity of peptides is shown in Table 1. The purity was obtained by calculating the percentage of peak area of each peptide. The purity of the peptides were further confirmed by LCMS and the values differed in 0.1–0.5% (Table 2). The completed coupling of each amino acid to form the peptide was observed through the Kaiser test (colour test), melting point, HPLC and LCMS analysis which provided the molecular weight of the peptides (Table 2).

**Table 1:** Physical characterizations and HPLC analysis of peptides containing histidine and aspartic acid. The solubilities of peptides were tested in water and confirmed by their charges at the iso-electric point (pI values)

Peptide	% Yield	Morphology	Solubility	Charge	pI	*R*_T_/min	% Purity
P1	74.0	White, sticky powder	Hydrophilic	0.1	7.57	42.6	81.7
P2	74.2	Yellow powder	Hydrophilic	0.1	7.57	24.0	84.1
P3	83.1	White powder	Hydrophilic	0.1	7.57	23.9	73.4
P4	69.9	White powder	Hydrophilic	0.1	7.88	7.24	99.5
P5	65.6	Pale yellow powder	Hydrophilic	0.1	8.26	7.69	99.2

**Table 2:** LCMS analysis of peptides containing histidine and aspartic acid

Peptide	Mol. weight^a^/gmol^−1^	Mol. weight^b^/gmol^−1^	*m*/*z*^c^	Charge (z)	*R*_T_/min	% Purity
P1	633.66	633.26	634.26	+1	4.969	81.4
P2	709.76	709.29	710.30	+1	5.754	84.0
P3	661.72	661.29	662.30	+1	5.259	72.9
P4	397.39	397.15	398.40	+1	7.242	99.0
P5	423.43	423.25	424.42	+1	7.690	98.9

a Calculated molecular weight.

b Experimental molecular weight.

c Mass/charge ratio obtained from MS analysis.

The calculated molecular weight tallies with the molecular weight of peptides from the instrument (Table 2). The purities of the peptides are similar when analysed by both HPLC and LCMS. The charges of the peptides were calculated to be +1 employing the *m*/*z* charge ratio, making the peptides suitable for binding of Cu(ii) ions. P4 & P5 peptides appear at a higher retention time as they were analysed using a different LCMS instrument under different conditions including different column (ESI Fig. 5†). CuP1–CuP5 were synthesized and purified as per the method discussed in Section 2.4.

### 3.3 Physicochemical characterization

The purity of the copper(ii)-peptides was determined by its melting point (decomposed) and the percent yields of the copper(ii)-peptides were fair. The yields of copper(ii)-peptides were compared with Stavropoulos *et al.* (1998) where copper(ii) was coordinated with histidine rich tetrapeptide in his research^16^ and similar yields were obtained in this research (refer to ESI†). The secondary structure analysis by CD (ESI Fig. 1 and Table 2†) and the complementary analysis of chirality rotation of peptides and copper(ii) peptides by polarimeter are also mentioned under ESI (Table 1†).

The copper bound peptides and their respective peptides were analysed further by spectral analysis. The data obtained is displayed in ESI Table 3† with a pictorial representation of UV-Vis spectroscopy results in ESI Fig 4.† The peptides were analysed in order to determine the location of copper(ii) binding to the peptide (side chain of amino acid), confirm the existence of copper by measuring the fluorescence emitted (Fig. 3) and to observe the shift in bands caused by copper(ii) and acetate molecules in the peptide by FTIR analysis.

**Fig. 2:**
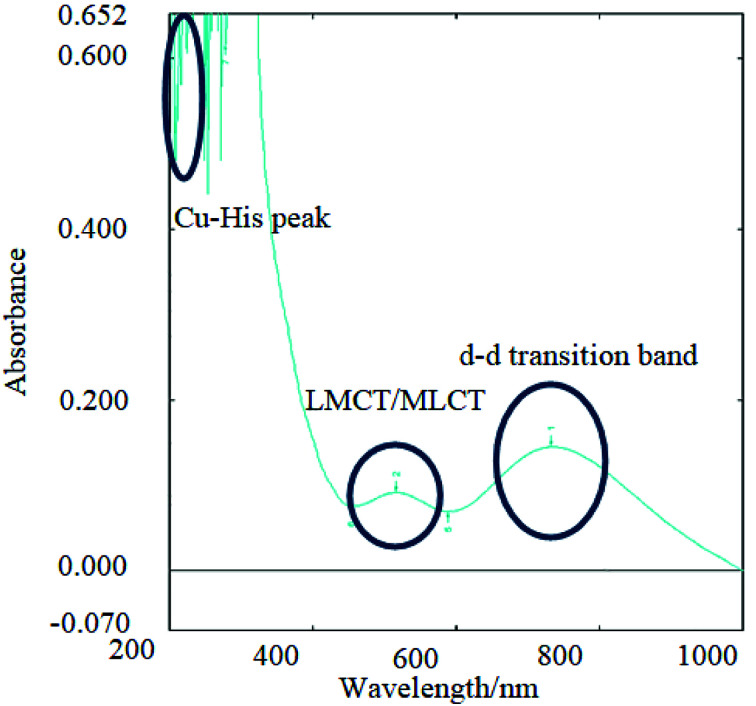
UV-Vis analysis of CuP4 where copper(ii) acetate is bound to P4 and the main assignments are shown herein. Imidazole ring in histidine of all peptides was detected at 230–260 nm (multiple peak signals due to resonance of C=N), charge transfer peak detected in the range of 500–600 nm for Cu(ii) to peptides and d–d transition band detected from 700–800 nm for the d^9^ electrons of Cu(ii) ions in peptides.^32^

**Fig. 3:**
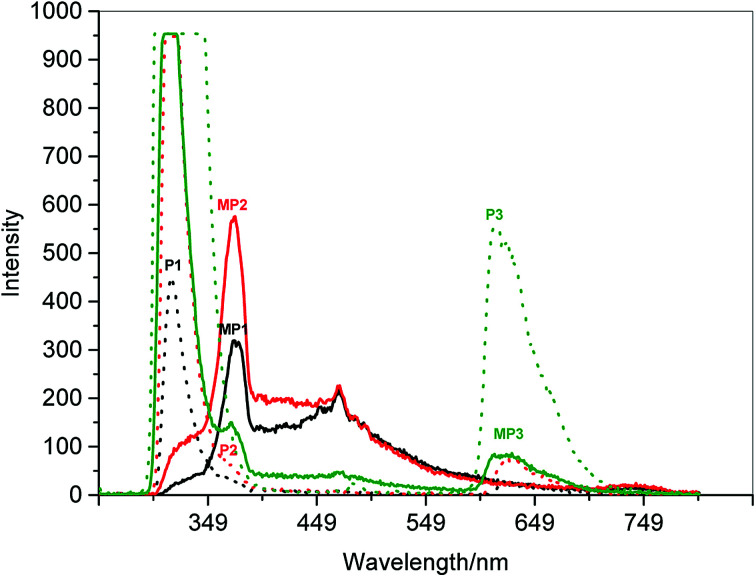
Fluorescence spectrum of the first three peptides and their Cu(ii)-peptides. The dotted lines represent the peptides while the solid lines of the same colour represent the respective copper(ii)-peptides; black: P1, red: P2 and green: P3. The fluorescence data complements the UV data for the detection of Cu(ii) ions bound to the imidazole ring bound as observed in the right shift and quenched peaks from 320 nm to 380 nm. Further quenching was observed for CuP3 at 620 nm.

Peptides have π bonds (C=O) and aromatic rings (like the side chain in histidine) that can be observed from the n–π* and π–π* (refer to ESI Table 3†). Similarly, these transitions can also be observed in copper-peptides indicating that copper binding does not affect the C=N and C=O in the transition state. The d–d transition and metal-to-ligand charge transfer (MLCT) bands were observed in the copper(ii)-peptides indicating the presence of a single d–d transition band that is characteristic of a d^9^ Cu(ii) ion as shown in Fig. 2.^20,31^

The results are in agreement with other reported data that d–d transitions of copper(ii)-tetrapeptides have absorption at the said wavelength with molar absorptivity in the range of about 2–3 L cm^−1^ mol^−1^ and the charge transfer bands (CT bands) for copper bound peptides normally have similar or lower molar absorptivity than that of d–d transition bands and appear in the range of 400–600 nm as was observed from CuP4 in Fig. 2.^24^ No charge transfer band and a weak d–d transition band was observed in CuP5 probably due to the low concentration of copper(ii)-peptide.

All the metal salts are expected to bind to the imidazole ring of histidine.^19^ This was observed in both the UV-Vis and UV-fluorescence spectra (ESI Fig 4 and 5†) where quenching of the peptide band at 620 nm was caused by the formation of new bonds and electrostatic force of attraction between copper(ii) and nitrogen in the imidazole ring of histidine or copper(ii) and COOH of the aspartic acid.^20^ Copper and zinc binds to the nitrogen (C=N) in the imidazole ring of histidine in the range of 250–300 nm giving rise to a strong sharp peak with high absorbance. However, it was also mentioned that copper also binds to the carboxylate side chain of aspartic acid and henceforth, the values are shifted further right (higher wavelength) due to the acidic nature of the copper(ii)-peptides, as observed in this study.^5,19^

No charge transfer band and only a weak d–d transition band were observed in CuP5 probably due to the low concentration of copper(ii)-peptide. Hence quenching was observed at 310 nm for CuP4 and CuP5 (refer to ESI†). This region indicates the copper binding to the nitrogen in the imidazole ring of histidine when compared to Mylonas (2004) research.^19^ A higher intensity peak was observed at 380 nm for CuP1-3 compared to their parent peptides indicating that presence of Fmoc in the copper(ii)-peptide affect the binding of copper(ii) to the peptide as opposed to CuP4 and CuP5.

Amide I are the strongest bands among all the amide bands in peptides and proteins. This band is due to the stretching vibrations of the C=O (∼85%) and C–N (∼10%). Meanwhile, amide II band is majorly formed from in-plane N–H bending (strong band) and stretching vibrations of the C–N and C–C (weak band).^19^ The observed data (ESI Table 3†) were in agreement with other reported data where the ranges of specific amide bands were obtained and through these results, it is concluded that the peptides followed the β-sheet structure^22,23^ which was also in agreement with the data from the Circular Dichroism studies (refer to ESI†). With the combination of UV-Vis and FT-IR results, there are two extra binding bands observed, indicating that 2 copper atoms were involved in the binding process as observed in ESI Fig. 2.†

In ESI Fig. 2,† the peptides and their copper peptides were compared using FTIR analysis. Two different bands were observed for each peptide at 1250 cm^−1^, indicating the free C=N in imidazole ring. These bands were not observed for all the copper(ii) complexes. Extra bands of C=O were observed at 1200–1100 cm^−1^ for the copper(ii) peptide complexes indicating the presence of acetate ions from copper(ii) acetate.

### 3.4 Catalysts in aldol reactions

The synthesized peptides and their copper-peptides were employed as catalysts in aldol reactions where an aldehyde and a ketone react together to give an aldol product. The purpose of the catalyst is to modify the rate of reaction in which the enantiomers of the aldol adduct can be separated.

**Scheme 2:**
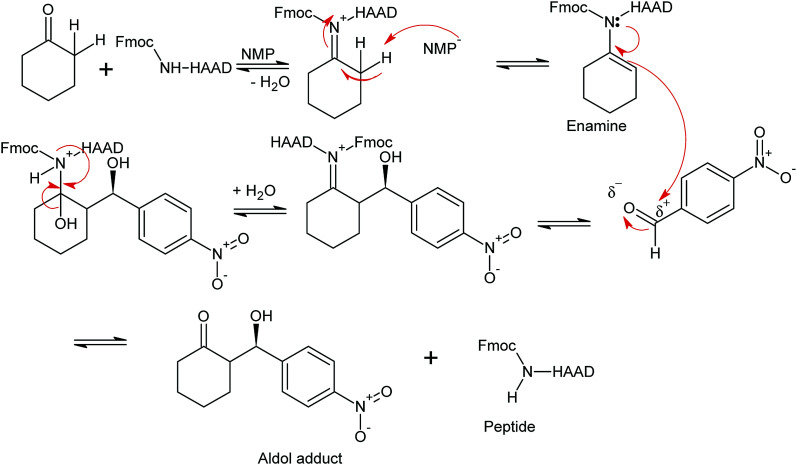
Mechanism of aldol reaction between *p*-nitrobenzaldehyde and cyclohexanone using P1 as catalyst. R^1^ = Fmoc or H, R = Peptide or copper(ii)-peptide sequence from *N*-terminal.^27^

**Scheme 3:**
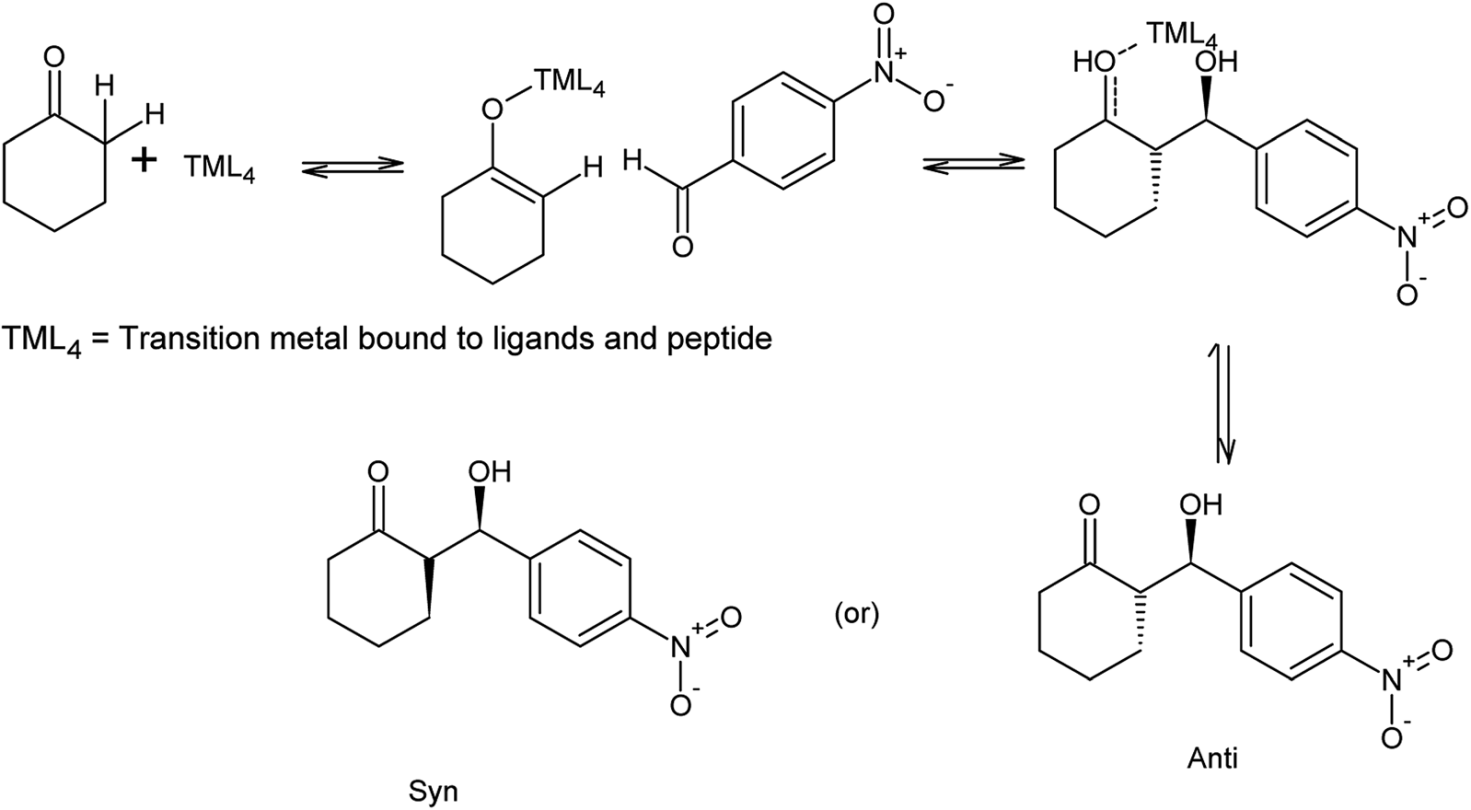
Mechanism of aldol reaction between *p*-nitrobenzaldehyde and cyclohexanone using copper-peptide as catalyst.^25,29^

Referring to the mechanism of aldol reaction (Scheme 2), the substrates bind to the *N*-terminal end of the peptide. It has been suggested that the mechanism of copper-peptides as catalysts are different from peptides.^25^ According to Juaristi *et al.* (2012) who studied transition metal bound to ligands as catalyst in aldol reactions, a mechanism was proposed (Scheme 3). *E*-enolate was preferred compared to *Z*-enolate and hence *anti* isomer was favoured. Based on the various characterizations of copper-peptides, it was also concluded that copper binds to the nitrogen of the imidazole ring in histidine. For the first three peptide sequences, histidine was placed at the *N*-terminal of the peptides. Theoretically, there will be less percent yield and percent ee of the aldol adduct when its copper-peptide is employed as catalyst due to steric hindrances. Hence histidine placed at the center of the tetrapeptides sequences was also designed (P4 & P5) and its copper-peptide synthesized (CuP4 & CuP5). Another aldol reaction between *p*-anisaldehyde (electron-donating group, –OCH_3_) and cyclohexanone was carried out as a comparison to the aldol reaction between *p*-nitrobenzaldehyde (electron withdrawing group, –NO_2_) and cyclohexanone (Table 3).

**Table 3:** % Yield and % ee of (*S*)-2-[(*R*)-hydroxy(4-nitrophenyl)methyl] cyclohexan-1-one and (*S*)-2-[(*R*)-hydroxy(4-methoxyphenyl)methyl] cyclohexan-1-one

Entry	Catalysts	(*S*)-2-[(*R*)-hydroxy(4-nitrophenyl)methyl] cyclohexan-1-one		(*S*)-2-[(*R*)-hydroxy(4-methoxyphenyl)methyl] cyclohexan-1-one	
		% Yield	% ee	% Yield	% ee
1	No catalyst^a^	67.2	0	1.1	0
2	Proline^b^	41.8	73.2	83.0	99.4
3	P1	53.8	22.2	21.5	0
4	CuP1	18.1	87.2	84.7	95.5
5	P2	66.5	22.9	25.8	0
6	CuP2	31.5	88.7	3.3	68.2
7	P3	41.9	94.4	84.4	97.5
8	CuP3	40.2	80.2	29.6	91.0
9	P4^c^	55.0	98.0	91.2	98.4
10	CuP4	50.5	0	22.1	0
11	P5	56.6	—	39.6	99.0
12	CuP5	50.7	0	21.2	94.4

a Negative control.

b Positive control.

c P4 was observed to be the best catalyst for both the aldol reactions, followed by P3. The copper-peptides (exceptional for CuP4) catalyzed both the aldol reactions with higher enantioselectivity (94–98%) and yield of products as compared to proline.

Based on the data shown in Table 3, there could be a different mechanism on how the substrates bound to copper-peptides. Instead of binding to the nitrogen at the *N*-terminal, they could bind to the transition metal, in this case, copper(ii) (3d^9^4s^0^). Aldol reaction, without any catalyst, generally produces an aldol product without any enantiomers. Proline was used as a positive control as a catalyst in an intermolecular aldol reaction as it has already been known for its good catalytic activity. Proline, when catalysing the aldol reaction between *p*-nitrobenzaldehyde and acetone gave 68% yield and 76% ee.^26^ It is also mentioned that aldol reactions employing aromatic aldehydes in the presence of any catalyst produced aldol adducts with moderate percent yields and percent ee.^26^ An oligo-peptide with the sequence of H-pro-glu-leu-phe-OH catalysed the aldol reaction between acetone and 4-nitrobenzaldehyde with 66% ee with 96% yield.^27^

The percent yield obtained in Table 3 was after the aldol products were separated from the excess cyclohexanone through column chromatography. All the copper bound to peptides subjected as catalysts had a lower yield than its own parent peptide. Copper bound to P1 and P2 gave a better separation of *anti* and *syn* enantiomers than its own parent peptides. Previously, as discussed by Zou *et al.* (2005), a dipeptide ala–ala gave a higher yield but lower percent ee as the time increased from 24 to 48 hours. This observation was also possible in this study as all the reactions were conducted for 48 hours.^28^ This was because, after 24 hours, the reaction was not completed by observing the TLC. This meant that steric hindrance was not a factor that affected the efficiency of catalyst in aldol reactions but it affected the yield of the aldol adducts unlike P3 that gave a higher yield and percent ee than its copper bound peptide. The percent yield and percent ee were comparable to a study by Bayat (2014) whose aldol adducts were catalysed by a 16 amino acid peptide and had >60% yield with >80% ee.^29^

On the other hand, the AGHD peptide (P4) produced aldol products with moderate yield and a very high percent enantioselectivity, more than any other peptide (percent ee = 98.0%). When compared to the use of dipeptide ala–gly (AG) as catalyst, the aldol adducts yielded 46% with percent ee of 81% over 48 hours.^28^ This meant that with the existence of histidine and aspartic acid, the percent yield and percent ee increases. Unlike PGHD (P5), there were no peaks obtained in the spectrum, though proline by itself is said to be an efficient catalyst in aldol reactions.^26^ Copper bound to P4 catalysed the reaction to produce one aldol product while copper bound to P5 gave no product at all.

The aldehyde *p*-anisaldehyde has a methoxy group that donate electrons into the benzene ring. The aldol reaction employing these two substrates is shown in Scheme 3. Previous research has shown that with the presence of the donating group, the percent ee of the *anti* and *syn* isomer is greatly reduced.^30^ This aldol reaction was carried out with the use of the same catalysts and conditions of the previous aldol reaction and the results are tabulated in Table 3. The melting point of the pure aldol products were measured and recorded as 160 °C. This value coincided with literature value of 159 °C.^25^ The mechanism of the reaction was the same as the mechanism of the aldol reaction between *p*-nitrobenzaldehyde and cyclohexanone (Schemes 2 and 3).

When compared to the first aldol reaction, the catalysts that gave a high percent ee for (*S*)-2-[(*R*)-hydroxy(4-nitrophenyl)methyl]cyclohexan-1-one also gave high yield and percent ee for (*S*)-2-[(*R*)-hydroxy(4-methoxyphenyl)methyl]cyclohexan-1-one. CuP3 and P5 gave a low yield of aldol product but with high enantioselectivity. These results were different from the data reported by Yilmaz *et al.* (2014) where with the use of the similar catalyst produced higher yields and percent ee for the *p*-nitrobenzaldehyde substrate with 95% yield and 90% ee compared to the *p*-anisaldehyde substrate with 40% yield and 50% ee.^30^

In another study by Juariasti (2012), dipeptides containing *S*-proline catalysed the aldol reaction between different substrates in solvent-free conditions similar to the aldol reactions carried out in this research. When *p*-nitrobenzaldehyde was introduced as the substrate with cyclohexanone, the percent yield and percent ee of (*S*)-2-[(*R*)-hydroxy(4-nitrophenyl)methyl]cyclohexan-1-one (89% and 91% respectively) were greater than the aldol adduct of *p*-anisaldehyde and cyclohexanone (51% and 50% respectively). It was also deduced that sulphur containing peptides gave higher enantiomeric selectivity of product than non-thiodipeptides, though the product formed was of lower yields.^25^

## 4. Conclusion

Five tetrapeptides were designed and synthesized with amino acids containing side groups that can bind to transition metals (histidine and aspartic acid). All the peptides were hydrophilic and had +1 charge using the *m*/*z* ratio from the LCMS data. The theoretical molecular weight matched the molecular weight attained by the mass spectrum. After determining their positive binding and the stability (constant secondary structure) through various spectroscopic techniques, these copper(ii)-peptides were purified and tested for their catalytic activity in aldol reactions.

P4 was observed to have the highest catalytic activity with the largest amount of the *anti* enantiomer adduct produced for both the reactions. High enantioselectivity but low yield was observed for most of the copper-peptides due to the steric hindrance caused by the bulky structure. However, the advantage of these copper peptides as catalyst were the presence of copper(ii) ions that not only increased the chemical and thermal stability of the peptide complexes as observed through CD analysis (ESI†) but also due to its bulky nature of copper(ii)-peptides, *anti* enantiomers were preferred for both the reactions.

## Conflicts of interest

The authors confirm that there are no known conflicts of interest associated with this publication and there has been no significant financial support for this work that could have influenced its outcome.

## Supplementary Material

RA-008-C8RA06814C-s001
