# Supplementary material for: Histidine-based copper tetrapeptides as enantioselective catalysts for aldol reactions
Source: RSC Adv. 2018 Oct 2;8(59):34004–11. doi: 10.1039/c8ra06814c (PMC9086720; doi:10.1039/c8ra06814c)
Supplement: RA-008-C8RA06814C-s001 [file RA-008-C8RA06814C-s001.pdf]

Electronic Supplementary Material (ESI) for RSC Advances.
This journal is © The Royal Society of Chemistry 2018

SUPPLEMENTARY INFORMATION

List	Caption
S.I. Table 1	Physical properties of peptides and their CuP.
S.I. Table 2	The secondary structures of peptides and their CuP analyzed at different temperatures.
S.I Table 3	UV and FTIR spectral analysis of peptides and their CuP.
S.I Table 4	Raw data of HPLC analysis of aldol adducts for Reaction 1 and 2.
S.I Scheme 1	SPPS Scheme of P1.
S.I. Figure 1	CD spectra of P1-P3 and their copper(II)-peptides analyzed at room temperature (25°C) indicating the secondary structures opted by their highest percentage as shown in S.I. Table 2.
S.I Figure 2	FTIR analysis of P1-P3 and CuP1-CuP3.
S.I. Figure 3	FTIR analysis of P4, CuP4, P5 and CuP5.
S.I. Figure 4	UV-Vis spectrum of P1-P5.
S.I. Figure 5	LC-MS of peptides P1-P5.
S.I. Figure 6	HPLC chromatograms of aldol reaction between *p*-nitrobenzaldehyde & cyclohexanone.

S.I. Table 1 Physical properties of their peptides and copper(II)-peptides listing the yield and purity in terms of percentage, melting/decomposing point and their chiral optical rotation value at room temperature (25°C).

P/CuP	% Yield	% Purity	Melting point/°C	$\alpha[D]^{25°C}$
**P1**	74.0	80.0	191.0 (m)	-12
**CuP1**	65.0		205.2 (d)	214
**P2**	74.2	84.0	210.0 (m)	-14
**CuP2**	56.0		220.6 (d)	85
**P3**	83.1	73.0	200.0 (m)	1
**CuP3**	53.3		208.5 (d)	93
**P4**	70.0	99.5	162.4 (m)	-10
**CuP4**	48.1		245.0 (d)	227
**P5**	65.6	99.2	130.0 (m)	-4
**CuP5**	49.6		210.6 (d)	460

m-melted d-decomposed

S.I. Table 2 The secondary structures of peptides and their CuP analyzed at different temperatures

**Peptides**	**Secondary structures**		
	4°C	25°C	60°C
**P1**	β-sheet (53.2%)	**β-sheet (52.2%)**	β-sheet (38.3%)
**CuP1**	Random (67.2%)	**Random (67.6%)**	Random (71.5%)
**P2**	Random (62.8%)	**β-sheet (82.9%)**	β-sheet (82.9%)
**CuP2**	Random (58.6%)	**Random (68.7%)**	Random (70.7%)
**P3**	Random (50.7%)	**Random (47.5%)**	Random (53.2%)
**CuP3**	β-turn (57.2%)	**Random (42.4%)**	Random (81.4%)
**P4**	β-sheet (40.9%)	**β-sheet (38.1%)**	β-sheet (75.8%)
**CuP4**	β-sheet (49.6%)	**α-helix (39.5%)**	β-sheet (96.3%)
**P5**	β-sheet (80.1%)	**β-sheet (40.2%)**	β-sheet (80.3%)
**CuP5**	β-sheet (53.4%)	**Random (38.3%)**	β-turn (43.9%)

S.I Table 3 UV spectral analysis of P1-P5 and copper bound to P1-P5 in compliment with the FTIR spectral analysis with the peak assignments

P/MP	UV Spectral Analysis			FTIR Analysis	
	UV-Visible λ/nm	log ε/ L·cm^{-1}·mol^{-1}	Assignment	Wavenumber (υ) (type of peak)/ cm^{-1}	Assignment
P1	π-π* transition at 200-270 nm & n-π* transition at 270-330 nm. (no peaks observed at 400-750 for all peptides and copper(II)-peptides) [31]			3277 (s,sh)	2° amine (3280-3250 cm^{-1})
				1251 (w,sh)	C-N (imidazole ring)
				1188 (s,sh)	C-O stretch of COOH
				1626 (s,sh)	Amide I & C=O bond
				1437 (w,b)<br>1538 (s,sh)	Amide II
P2				3277 (s,sh)	2° amine
				1250 (w,sh)	C-N (imidazole ring)
				1180 (s,sh)	C-O stretch of COOH
				1628 (s,sh)	Amide I & C=O bond
				1434 (w,b)<br>1540 (s,sh)	Amide II
P3				3279 (s,sh)	2° amine
				1255 (w,sh)	C-N (imidazole ring)
				1183 (s,sh)	C-O stretch of COOH
				1628 (s,sh)	Amide I & C=O bond
				1433 (w,b)<br>1537 (s,sh)	Amide II
P4				3298 (s,sh)	1° amine (3400-3280 cm^{-1})
				1266 (w,sh)	C-N (imidazole ring)
				1133 (s,sh)	C-O stretch of COOH
				1659 (s,sh)	Amide I & C=O bond
				1431 (w,sh)<br>1546 (s,sh)	Amide II
P5				3318 (s,sh)	1° amine
				1138 (s,sh)	C-O stretch of COOH
				1650 (s,sh)	Amide I & C=O bond
				1431 (w,sh)<br>1522 (s,sh)	Amide II
CuP1	714	2.04	d-d transition	All the bands observed for peptides were observed here. Another amide II band was observed around 1460 cm^{-1} (s,sh) for all the copper peptides. Another peak (s,b) was observed in the range 3400-3600 cm^{-1} due to a combination of the 2° amine and acetate (COO) from the copper(II) acetate [25].<br>s-strong, w- weak, sh-sharp, b-broad	
	458	1.98	charge transfer		
	239	3.84	Cu-N=C imidazole		
CuP2	708	1.60	d-d transition		
	530	1.72	charge transfer		
	268	3.74	Cu-N=C imidazole		
CuP3	737	2.17	d-d transition		
	648	2.23	charge transfer		
	270	3.85	Cu-N=C imidazole		
CuP4	747	2.31	d-d transition		
	518	1.25	charge transfer		
	307	2.60	Cu-N=C imidazole		
CuP5	749	1.16	d-d transition		
	264	1.93	Cu-N=C imidazole		

S.I. Table 4 HPLC CHIRALCAK column analysis of aldol enantiomers from Reaction 1 and Reaction 2 with different catalysts

**Catalyst**	**(S)-2-[(R)-hydroxy(4-nitrophenyl)methyl] cyclohexan-1-one**				**(S)-2-[(R)-hydroxy(4-methoxyphenyl)methyl] cyclohexan-1-one**			
	R_TA/min	R_TB/min	% Area A	% Area B	R_TA/min	R_TB/min	% Area A	% Area B
**No catalyst[a]**	10.2	-	97.1	-	7.50	-	1.24	-
**Proline[b]**	10.3	14.9	86.6	13.4	7.50	10.4	62.0	0.190
**P1**	10.9	15.3	3.30	2.10	7.50	-	13.7	-
**CuP1**	10.8	14.5	35.2	2.40	7.46	10.2	85.9	1.98
**P2**	10.6	14.7	10.6	16.9	7.50	-	3.41	-
**CuP2**	10.5	14.5	80.5	4.80	7.50	10.2	7.03	1.33
**P3**	10.7	14.6	68.2	1.98	7.50	10.2	11.1	0.140
**CuP3**	10.6	14.4	18.5	2.03	7.50	10.2	9.14	0.430
**P4**	10.7	14.4	75.2	0.750	7.49	10.2	32.9	0.260
**CuP4**	10.5	-	96.0	-	7.50	-	1.60	-
**P5**	No product peaks at 10-15 mins				7.49	10.3	87.2	0.420
**CuP5**	10.7	-	16.08	-	7.49	10.3	17.0	0.490

%ee= [(Peak Area A (R) – Peak Area B (S))/(Peak Area A (R) + Peak Area B (S))]*100

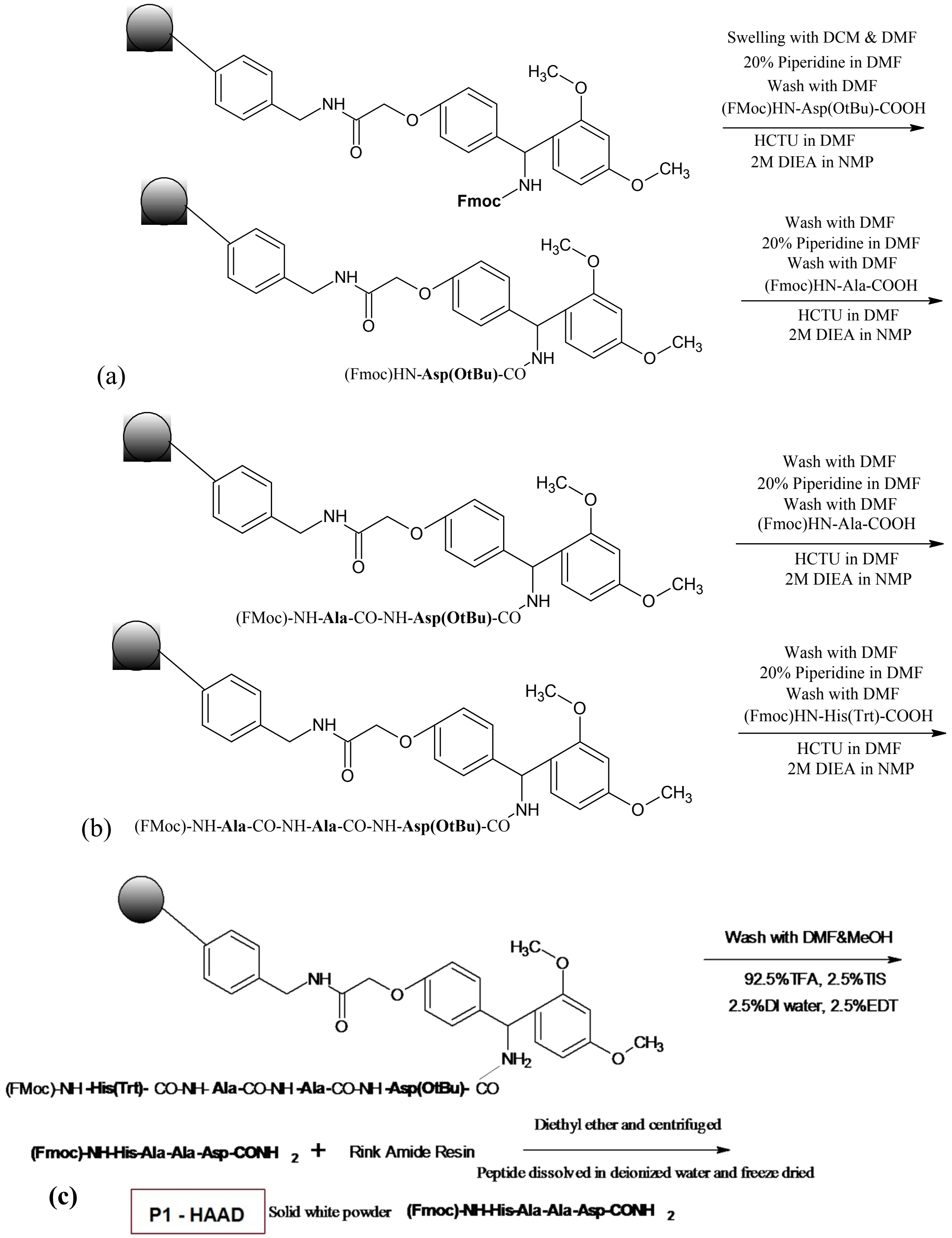

S.I. Scheme 1 SPPS scheme of P1; (a) Fmoc deprotection of rink amide resin and coupling of 1st amino acid from C-terminal; (b) Fmoc deprotection of 1st amino acid and coupling of 2nd and 3rd amino acid; (c) Fmoc deprotection of 2nd and 3rd amino acid and coupling of final (4th) amino acid; (d) Cleaving peptide from resin using cleavage cocktail mixture.

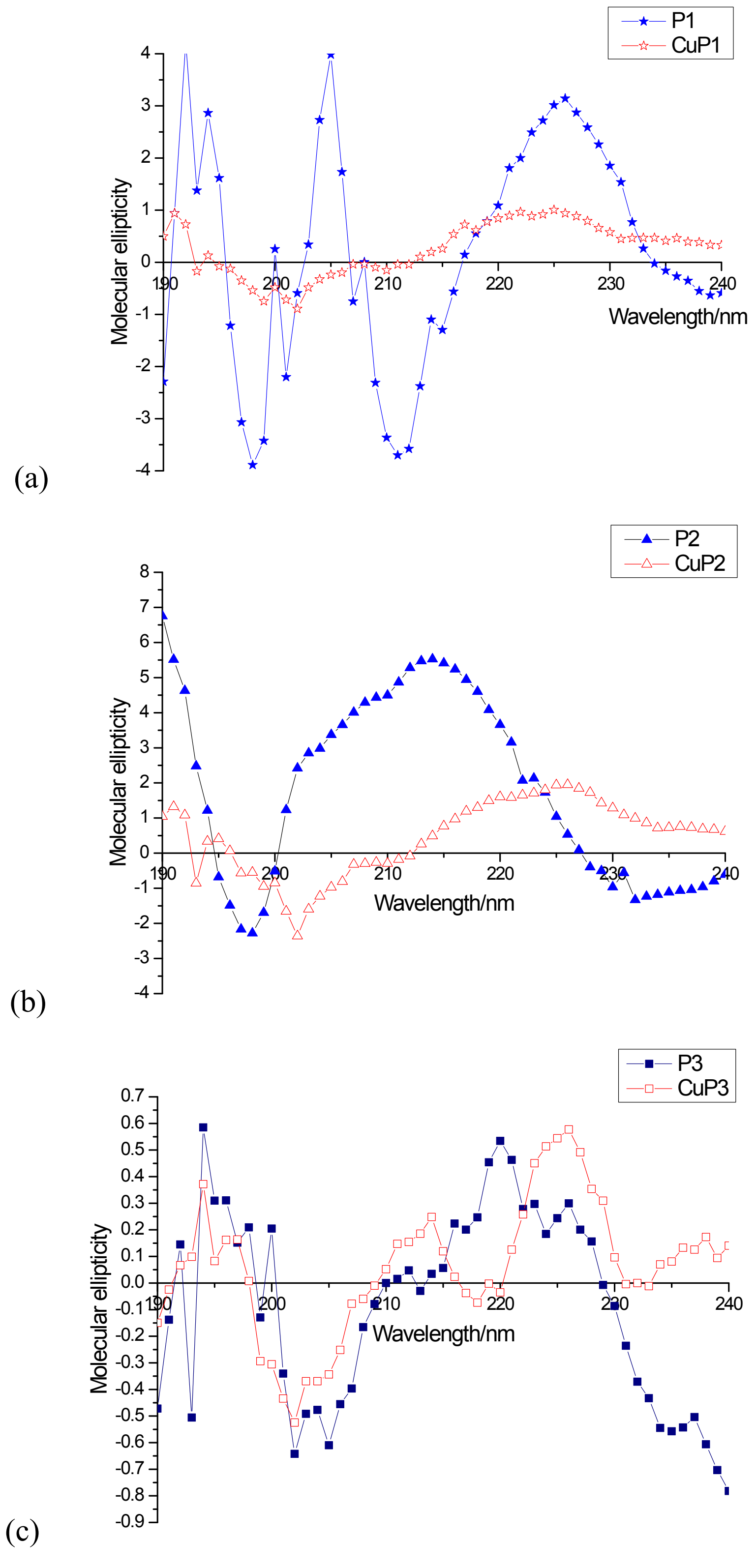

S.I. Figure 1 CD spectra of (a) P1 & CuP1 (5 mM, 400 µL), (b) P2 & CuP2 (5 mM, 400 µL) and (c) P3 & CuP3 (5 mM, 400 µL) analyzed at room temperature (25°C), their secondary structures opted by their highest percentage as shown in S.I. Table 2.

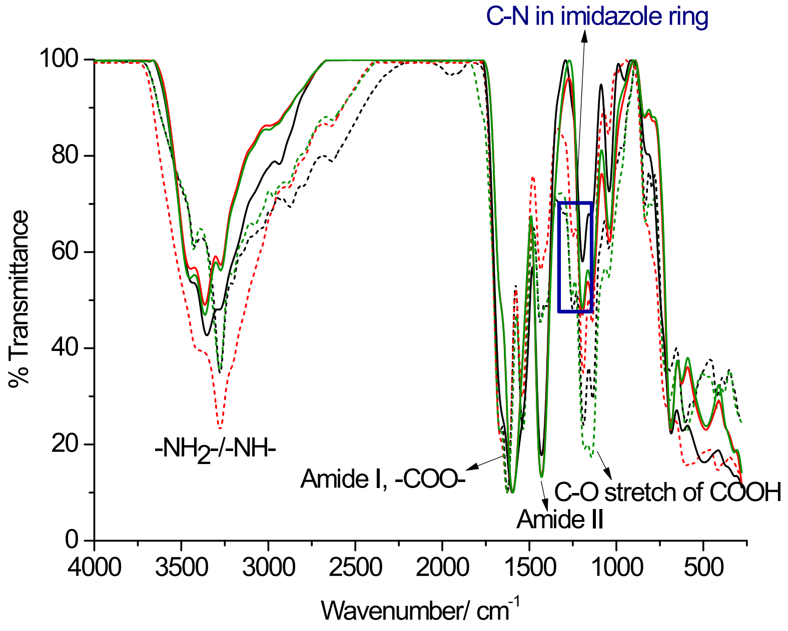

S.I. Figure 2 FTIR analysis of the P1-P3 and CuP1-CuP3. The dotted lines represent the peptides while the solid lines of the same colour represent the respective copper(II)-peptides; black: P1, red: P2 and green: P3. Most of the major peaks as assigned in the graph were shifted to the right for copper-peptides as compared to their parent peptides (in the range of 2000-500 cm^{-1}). The amide peaks (terminal end) of copper(II) peptides were shifted to the left when compared to their peptides (3400-3200 cm^{-1}).

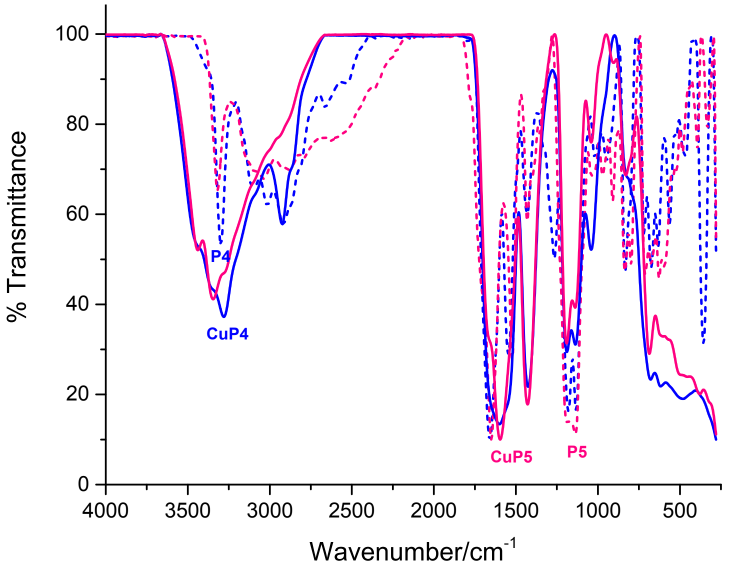

S.I. Figure 3 FTIR analysis of P4, CuP4, P5 and CuP5. The dotted lines represent the peptides while the solid lines of the same colour represent the respective copper(II)-peptides; blue: P4 and pink: P5.

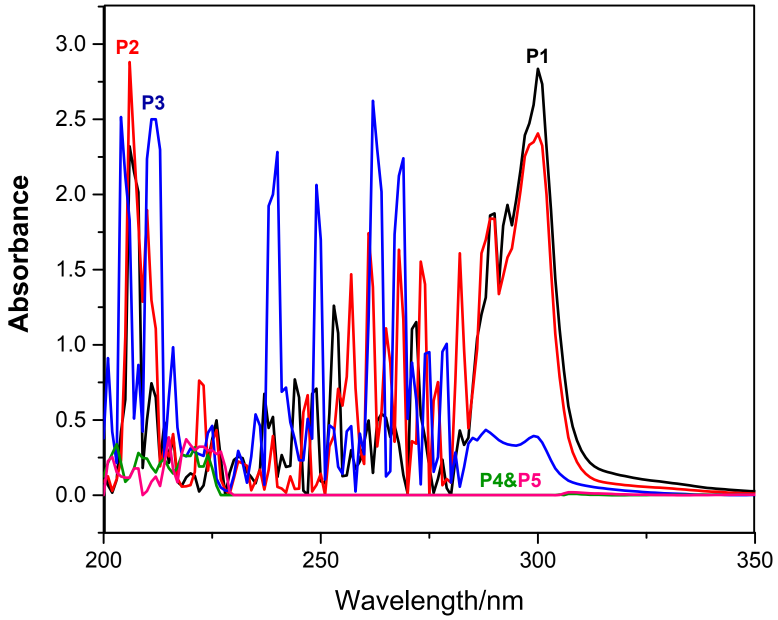

S.I. Figure 4 UV-Vis spectrum of P1-P5. 0.1M of P1-P3 are denoted by black, red and blue dotted lines, respectively, where as 0.01 M of P4 and P5 are denoted by green and pink dotted lines, respectively. The transitions that include n-π* and π- π* (peptides) appear around 270- 330 nm and 200-270 nm respectively. These transitions occur due to the presence of double bonds, cyclised rings and aromatic rings of the amino acids and amide bonds [21]. Hence they appear as several peaks clustered together as observed in the spectra of peptides.

(a)

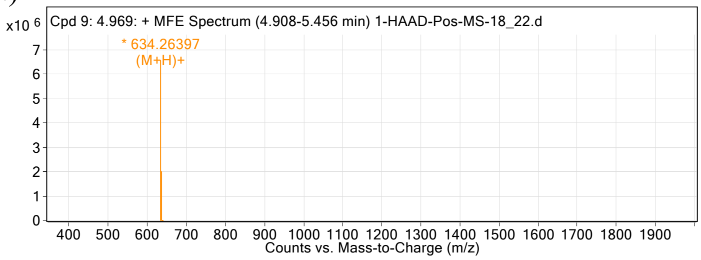

Compound Label	*m/z*	RT	Algorithm	Mass
HAAD Fmoc	634.26397	4.969	Find by Molecular Feature	633.25686

(b)

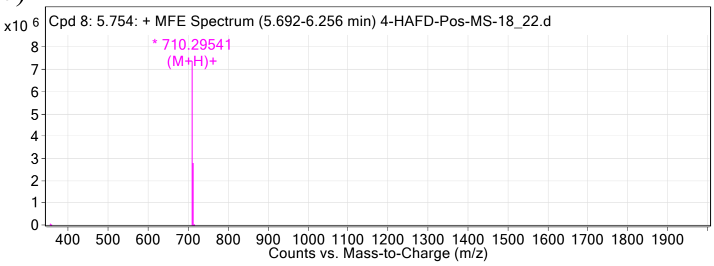

Compound Label	*m/z*	RT	Algorithm	Mass
HAFD w Fmoc	710.29541	5.754	Find by Molecular Feature	709.28869

(c)

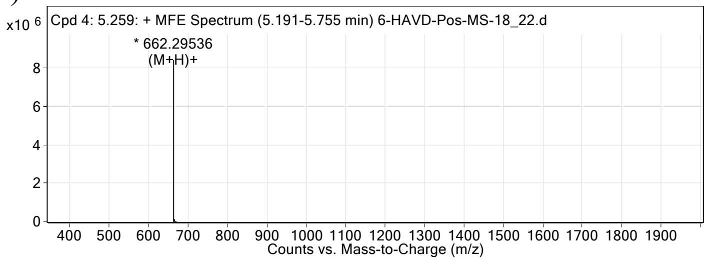

Compound Label	*m/z*	RT	Algorithm	Mass
HAVD Fmoc	662.29536	5.259	Find by Molecular Feature	661.28848

(d)

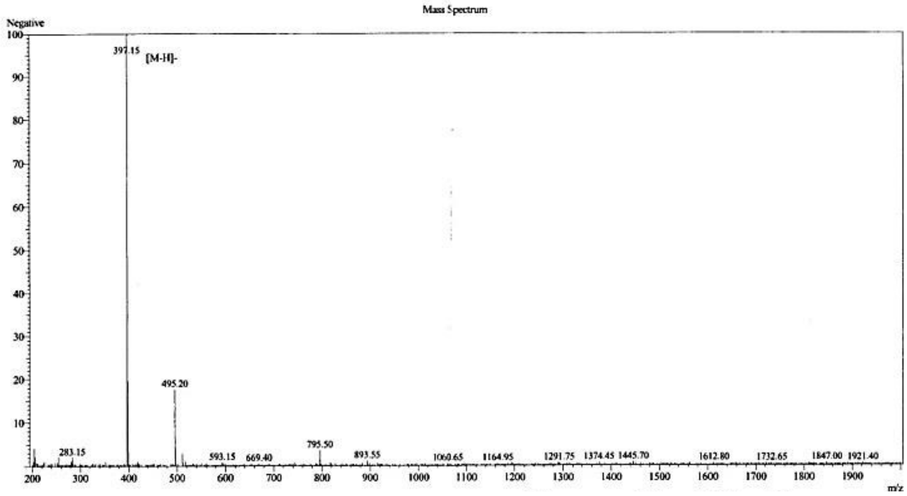

Compound Label	*m/z*	RT	Algorithm	Mass
AGHD w/o Fmoc	398.4032	7.242	Find by Molecular Feature	397.1503

(e)

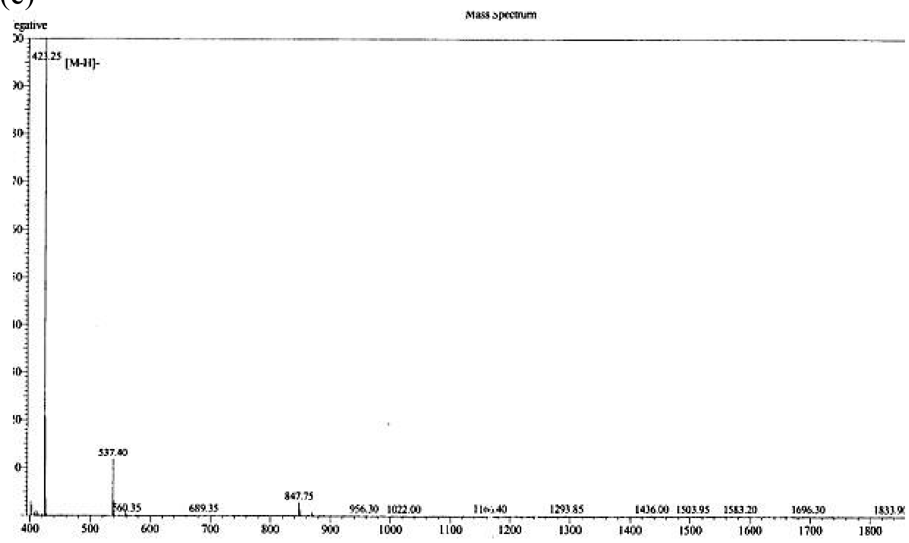

Compound Label	*m/z*	RT	Algorithm	Mass
PGHD wo Fmoc	424.42	7.690	Find by Molecular Feature	423.2513

S.I. Figure 5 LC-MS of peptides P1-P5.

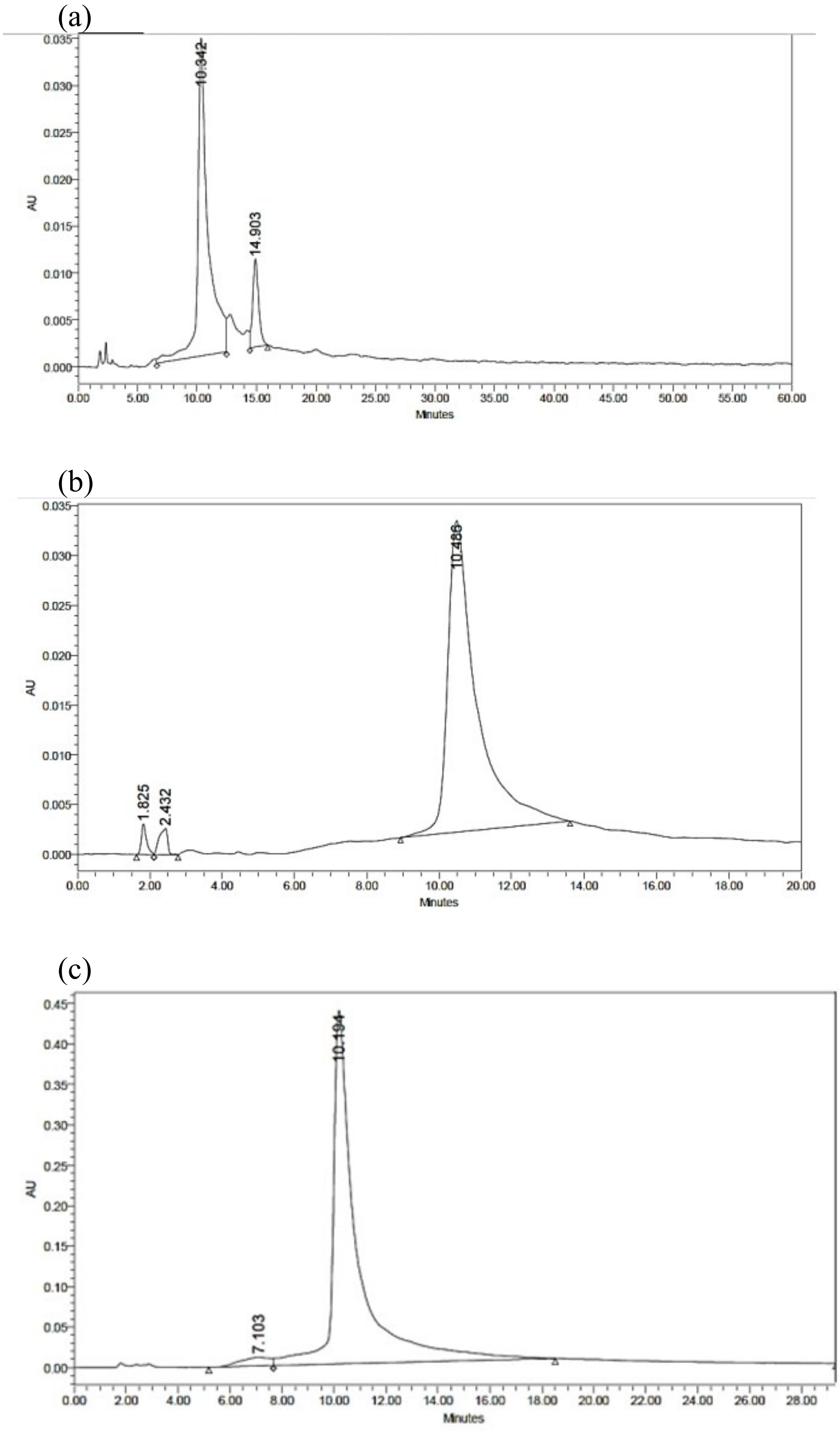

S.I. Figure 6 HPLC chromatograms of aldol reaction between p-nitrobenzaldehyde & cyclohexanone with catalysts (a) Proline (b) CuP4 (c) No catalyst.
